# High-performance photodetector based on an interface engineering-assisted graphene/silicon Schottky junction

**DOI:** 10.1038/s41378-021-00332-4

**Published:** 2022-01-07

**Authors:** Peirui Ji, Shuming Yang, Yu Wang, Kaili Li, Yiming Wang, Hao Suo, Yonas Tesfaye Woldu, Xiaomin Wang, Fei Wang, Liangliang Zhang, Zhuangde Jiang

**Affiliations:** 1 State Key Laboratory for Manufacturing Systems Engineering, Xi’an Jiaotong University, Xi’an, 710049 China; 2 MOE Key Laboratory for Nonequilibrium Synthesis and Modulation of Condensed Matter, Xi’an Jiaotong University, Xi’an, 710049 China

**Keywords:** Nanoscale devices, Optics and photonics

## Abstract

Graphene/silicon Schottky junctions have been proven efficient for photodetection, but the existing high dark current seriously restricts applications such as weak signal detection. In this paper, a thin layer of gadolinium iron garnet (Gd_3_Fe_5_O_12_, GdIG) film is introduced to engineer the interface of a graphene/silicon Schottky photodetector. The novel structure shows a significant decrease in dark current by 54 times at a −2 V bias. It also exhibits high performance in a self-powered mode in terms of an I_light_/I_dark_ ratio up to 8.2 × 10^6^ and a specific detectivity of 1.35 × 10^13^ Jones at 633 nm, showing appealing potential for weak-light detection. Practical suitability characterizations reveal a broadband absorption covering ultraviolet to near-infrared light and a large linear response with a wide range of light intensities. The device holds an operation speed of 0.15 ms, a stable response for 500 continuous working cycles, and long-term environmental stability after several months. Theoretical analysis shows that the interlayer increases the barrier height and passivates the contact surface so that the dark current is suppressed. This work demonstrates the good capacity of GdIG thin films as interlayer materials and provides a new solution for high-performance photodetectors.

## Introduction

Photodetectors with high detectivity and large on-off ratios have urgent market demand in the field of weak-signal detection, remote sensing, and optical communications^1–3^. Graphene, a typical representative 2D material, has ultrahigh carrier mobility at room temperature, a broadband absorption spectrum, and a flexible band structure, holding attractive advantages over conventional semiconductor materials^4,5^. Benefiting from its natural planar membrane-like structure, low-cost Schottky junctions are conveniently constructed when transferring graphene onto n-type silicon, which generates photocarriers based on the photovoltaic effect^6,7^. Previous studies have found that graphene/silicon (Gr/Si) Schottky photodetectors usually have difficulty providing both high responsivity and detectivity since the detectivity is mainly limited by the dark current, which closely depends on the contact interface and the Schottky barrier height^8^.

Recently, the insertion of a thin insulating oxide layer at the interface has been demonstrated to be effective in engineering the Schottky junction and suppressing dark current^9^. Li et al. first utilized a natural oxide layer (SiO_2_) as an interlayer^10^, which reduced the dark current from 9.35 nA to 0.1 nA, but the inevitable ever-growing thickness would block the tunneling of photogenerated carriers soon afterward. In addition, some solution-based interlayers have been proposed, but they normally suffer from nonuniform coating and an uncontrollable thickness^11^. In our previous work, graphene oxide flakes were inserted into the Gr/Si Schottky photodetector, which lowered the dark current by more than 10 times but encountered instability issues^12^. Therefore, the high dark current problem in Gr/Si Schottky junctions has never been completely resolved, and finding homogeneous and stable interlayer materials has profound effects on constructing high-performance photodetectors.

Gadolinium iron garnet (Gd_3_Fe_5_O_12_, GdIG) is a transparent ferromagnetic insulator with a garnet structure. It is a high-k material with a considerable dielectric constant (approximately 11.9)^13^ and superior insulating properties even at a very thin thickness, effectively minimizing the device size. The thickness and uniformity can be precisely controlled by magnetron sputtering technology. In addition, good temperature and chemical stability toward water vapor and oxygen enable the device to maintain long-term operation. Moreover, its natural magneto-optical modulation properties may hold the prospect of polarized photodetection for specific wavelengths^14^. These characteristics bode well for its appealing potential for the interface engineering of Gr/Si Schottky photodetectors.

In this paper, a graphene/GdIG/Si hybrid construction is proposed and fabricated for the first time. The homogeneous GdIG thin film acts as an interlayer to engineer the conventional graphene/silicon Schottky junction. A larger Schottky barrier and smaller reverse saturation current are observed. The high permittivity and controlled thickness help to suppress the dark current by 54 times at a −2 V bias. Photoresponse characterizations reveal that the Gr/GdIG/Si photodetector exhibits high performance in the self-powered mode in terms of an I_light_/I_dark_ ratio up to 8.2 × 10^6^, a specific detectivity of 1.35 × 10^13^ Jones, and a speed of 0.15 ms at 633 nm. In addition, the device shows environmental stability after several months in air. This study demonstrates the good capacity of GdIG as an interlayer material and provides a new approach for high-performance photodetectors.

## Materials and methods

**Fig. 1:**
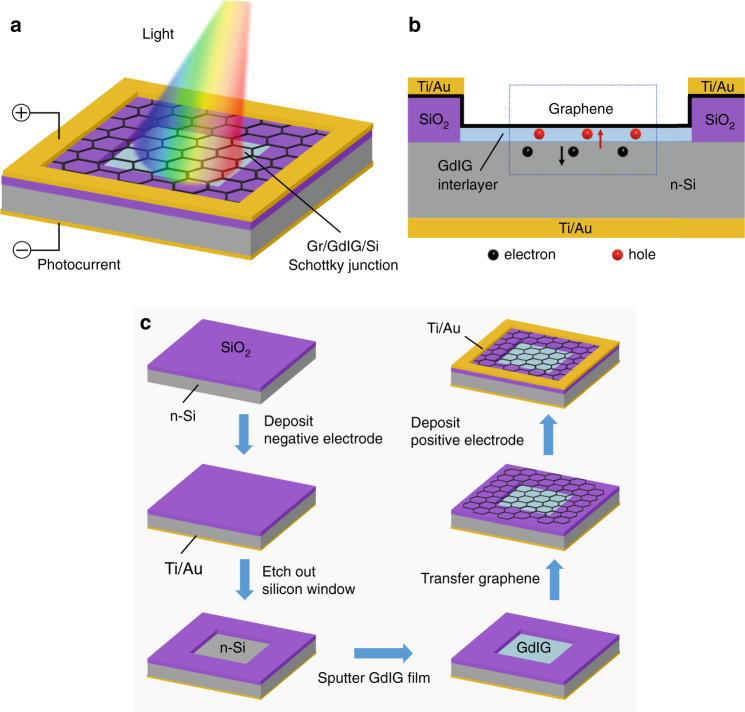
Device model and fabrication flow. **a** 3D schematic view and **b** Cross-section of the proposed Gr/GdIG/Si photodetector. **c** Schematic illustration of the fabrication process of the Gr/GdIG/Si photodetector

Figure 1a illustrates the schematic of this novel device, in which a GdIG thin film is deposited on an exposed silicon window via RF magnetron sputtering technology. The hybrid architecture is divided into two parts: the upper layer is graphene, and the lower layer is n-type silicon (n-Si). The contact interface between graphene and silicon forms a Schottky junction, and the GdIG thin film acts as the interlayer to facilitate carrier transmission, as shown in Fig. 1b. Here, graphene plays a role in both constructing Schottky junctions and collecting carriers as transparent electrodes.

The device was fabricated using a lightly doped n-type silicon (100) wafer with 300 nm SiO_2_ to load the top electrode. As illustrated in Fig. 1c, the natural oxide layer on the backside of the substrate was first removed using the buffered oxide etch solution, and a Ti/Au (20 nm/80 nm) bottom electrode was then immediately deposited by e-beam vapor deposition, forming ohmic contact with the silicon substrate. Next, a square junction area was defined via UV lithography on the front side, and SiO_2_ was etched away to expose the underlying silicon. The GdIG thin film was then deposited on the n-type silicon window, forming an interfacial layer. After that, the CVD-grown graphene film was transferred on the GdIG-coated surface to build a Gr/GdIG/Si Schottky junction. Finally, UV lithography and e-beam vapor technologies were used again to define and deposit the Ti/Au (20 nm/80 nm) top electrode. The fabrication is compatible with the conventional semiconductor process, which shows promise for building wafer-scale photodetector arrays. The detailed material synthesis methods are summarized in the Supplementary Information.

## Results and discussion

### Material and device characterization

**Fig. 2:**
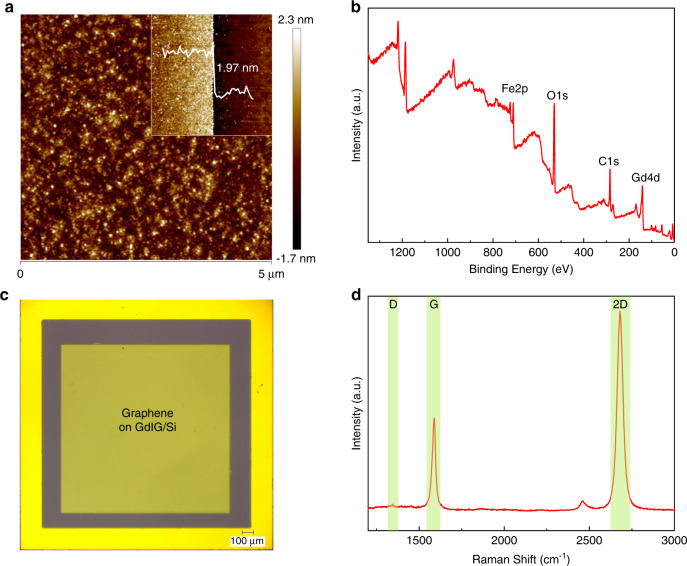
Characterization of the proposed Gr/GdIG/Si photodetector. **a** Surface morphology and thickness (inset) of the as-prepared GdIG film. **b** XPS spectrum of the GdIG film. **c** Optical image of the graphene transferred on GdIG coated n-type silicon, forming a square photosensitive area. **d** Raman spectrum of the monolayer graphene

Atomic force microscopy (AFM) was applied to assess the quality and large-area uniformity of the interlayer. Figure 2a shows the continuous and smooth GdIG film with a scanned area of 5 × 5 μm^2^, and the arithmetic mean roughness is 0.40 nm. The inset figure reveals a GdIG thickness of 1.97 nm, and the step sample used comes from the same batch. Surface chemical analysis of the GdIG film was carried out using X-ray photoelectron spectroscopy (XPS), as presented Sin Fig. 2b. The overview spectrum reveals the presence of Gd, Fe, and O elements. The core-level spectra of Gd 4d and Fe 2p are plotted in Fig. S1, from which we confirm the chemical formula. Figure 2c displays the smoothing of the transferred graphene on the square photosensitive area without folding and breakage. The structure was further investigated through Raman spectroscopy. As shown in Fig. 2d, the D, G, and 2D peaks with sharp Lorentz patterns are located at 1345 cm^−1^, 1588 cm^−1^, and 2679 cm^−1^, respectively. The very small D-peak indicates few defects, and the full-width half maximum of the 2D peak is calculated to be 36 cm^−1^. Meanwhile, the ratio of I_2D/G_ is 2.1, which indicates that the as-prepared graphene film is a high-quality monolayer.

### Photoresponse mechanism after insertion of GdIG

**Fig. 3:**
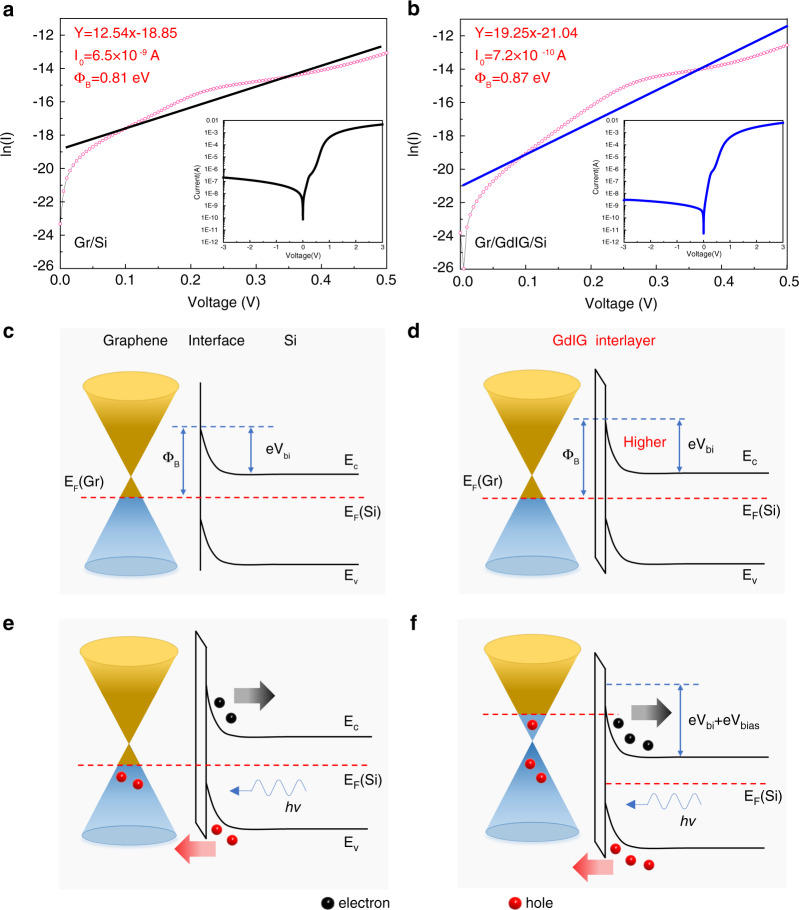
Photoresponse mechanism analyses. **a** Semi-log I-V curve (inset) and its linear fitting of Gr/Si Schottky junction to extract the parameters. **b** Semi-log I-V curve (inset) and its linear fitting of Gr/GdIG/Si Schottky junction. Energy band diagrams of **c** Gr/Si junction under dark condition, **d** Gr/GdIG/Si junction under dark condition, **e** Gr/GdIG/Si junction under illumination and **f** Gr/GdIG/Si junction under illumination with a reverse bias voltage. Here, Φ_B_, V_bi_, e, E_C_, E_V_, E_F_(Gr), E_F_(Si), and V_bias_ represent the Schottky barrier height, built-in electric field, electronic charge, conduction band edge, valence band edge, Fermi level of Gr, Fermi level of Si, and the reverse bias voltage, respectively. The arrows indicate the direction of movement

According to thermionic emission theory^15^, the electrical transport properties of a Schottky diode inserted with an interfacial oxide layer can be described by

1 $$\begin{array}{l}I = I_0\left( {e^{\frac{{qV}}{{\eta kT}}} - 1} \right)\\\,\,\,\, = AA^{\ast} T^2e^{ - \sqrt \chi \delta }e^{ - \frac{{{{\Phi }}_B}}{{kT}}}\left( {e^{\frac{{qV}}{{\eta kT}}} - 1} \right)\end{array}$$

where *I*_*0*_ is the reverse saturation current. *q*, *η*, *k*, *T*, *A*, A*, and *Φ*_*B*_ are the electronic charge, ideality factor, Boltzmann constant, temperature, photosensitive area, Richardson coefficient, and Schottky barrier height, respectively. *χ* and *δ* represent the average barrier height and the thickness of the inserted film. The transmission factor across the interlayer can be depicted by the term $e^{ - \sqrt \chi \delta }$, indicating that the interlayer helps to reduce the reverse saturation current, which is the main component of the dark current. To verify the mechanism after GdIG insertion, the photovoltaic behavior of the Schottky junctions with and without the GdIG interlayer was tested. Two groups of ten samples were fabricated using the same process, among which five contained interlayers. The results for each group were comparable (presented in Section 5 of the Supplementary Information); thus, we selected a pair for in-depth study. The insets of Fig. 3a, b plot the semilogarithmic current-voltage (I–V) curves of the devices, from which typical rectifying properties can be observed. The Schottky diode parameters can be extracted from their linear fittings as described in Section 6 of the Supplementary Information. As shown in Fig. 3a,b, the ideality factor and reverse saturation current are extracted to be 3.1 and 6.5 × 10^−9 ^A for the Gr/Si photodetector and 2.0 and 7.2 × 10^−10 ^A for the Gr/GdIG/Si photodetector. In addition, the Schottky barrier heights are calculated to be 0.81 eV and 0.87 eV. Taken together, these results suggest that the inserted GdIG interlayer can suppress the reverse saturation current and increase the Schottky barrier height, making the junction closer to the ideal Schottky diode so that the ideality factor becomes lower.

As a crosscheck, band theory is adopted to interpret the photoresponse mechanism. A Schottky junction is constructed automatically when semi-metallic graphene and n-Si contact each other, leading to the formation of the built-in electric field (eV_bi_) and the rectifying Schottky barrier (Φ_B_) on the contact surface, as shown in Fig. 3c. However, due to the inevitable dangling bonds of graphene and the nonideal surface of silicon, an actual Gr/Si Schottky junction usually has a high density of surface states, which results in large carrier recombination. In addition, the lower barrier height enables thermally excited electrons to cross the interface, forming a reverse saturation current. After insertion of the insulating GdIG interlayer, graphene and silicon are spatially separated, naturally improving Φ_B_ and eV_bi_, as shown in Fig. 3d. Thermally generated carriers that intend to cross the barrier are blocked so that the reverse saturation current is suppressed. In addition, the homogeneous interlayer with fewer structural defects helps to passivate the interface and reduce the density of surface states, thus decreasing the recombination current. When the junction is irradiated, photogenerated carriers are produced and separated by the built-in electric field. The holes are injected into the valence band of graphene, while the electrons move into silicon, which subsequently leads to a photocurrent, as shown in Fig. 3e. Moreover, the insertion of a GdIG thin film allows photogenerated hole tunneling to graphene^16^, and the impact ionization effect during the tunneling process functions as photogain to increase the photocurrent^17^. When a reverse bias voltage (V_bias_) is applied externally, graphene opens up more accessible states for photoexcited holes, hence resulting in a larger photocurrent, as shown in Fig. 3f.

In general, the stronger Schottky barrier and lower surface states resulting from the GdIG interlayer help to facilitate the control of carrier transport.

### Performance of Gr/GdIG/Si Schottky photodetector

**Fig. 4:**
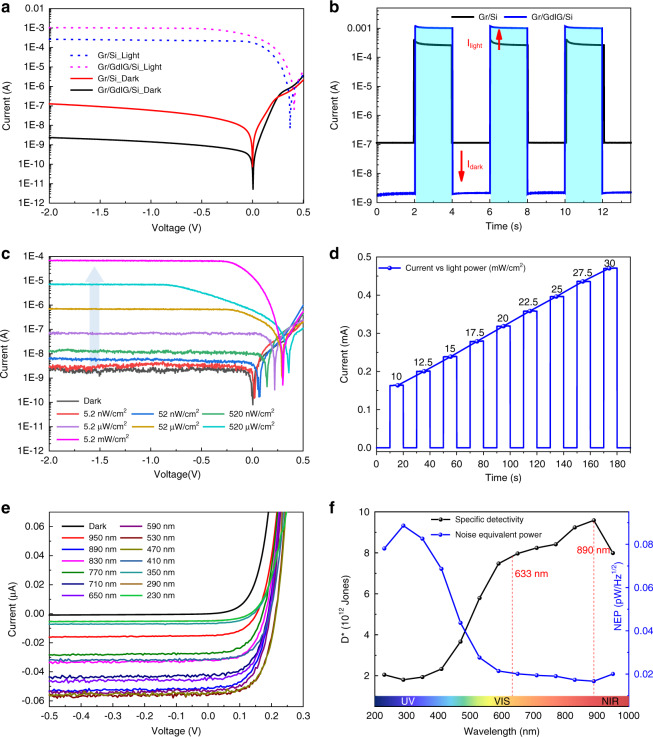
Photoresponse, power-dependent, and spectrum-dependent characteristics of the Gr/GdIG/Si photodetector. **a** Comparison of I-V curves between the photodetectors with and without GdIG interlayer. **b** Photocurrent versus time characteristics, showing the enhanced I_light_/I_dark_ ratio. **c** Photoresponse at large-scale power density variation (down to nW/cm^2^ level, demonstrating the weak-light detectability). The arrow indicates the direction of power increase. **d** Photoresponse with small steps of power variation at high intensities, indicating a near-linear behavior. **e** Multiband response at different incident wavelengths. **f** Specific detectivity and noise equivalent power from UV to NIR spectrum

It is now understood that GdIG plays an important role in modifying the Gr/Si Schottky junction, which is beneficial for high-performance photodetection. Therefore, the optical response of the Gr/GdIG/Si Schottky photodetector was measured and compared with the conventional construction. Figure 4a illustrates the I-V curves at room temperature under dark and 633 nm illumination with a power density of 60 mW/cm^2^. At an operating voltage of −2 V, the dark current of the proposed device is measured to be 2.35 nA, which is 54 times lower than that of the Gr/Si photodetector; meanwhile, the photocurrent increases from 0.26 mA to 1.04 mA so that the light/dark current ratio (I_light_/I_dark_), a critical parameter appraising the device signal-to-noise resolution, is as high as 4.4 × 10^5^ (Fig. 4b). The responsivity of the improved photodetector is 0.90 A/W. In addition, the impressive specific detectivity (D*) and noise equivalent power (NEP) of the Gr/GdIG/Si photodetector are 5.25 × 10^12^ Jones and 0.03 pW/Hz^1/2^, respectively. The proposed hybrid device displays a significant enhancement over the conventional structure, which is ascribed to the effective suppression of dark current and increase in photocurrent in the presence of an interfacial GdIG layer. It is worth mentioning that the present detectors can also work without an external bias (0 V) and display good self-power behavior. According to the photodiode mechanism, a built-in photovoltage arises at the junction interface when irradiated, which acts as a bias voltage to facilitate the transport of photogenerated carriers^18^. For the studied devices, the open-circuit voltage and the short-circuit current observed from I-V measurements increase from 0.37 V to 0.41 V and 177 μA to 367 μA, respectively, indicating that the photovoltaic and self-driven properties are also improved after the insertion of the GdIG interlayer. Further calculations suggest that in zero-biasing mode, the fascinating I_light_/I_dark_ ratio and specific detectivity of the Gr/GdIG/Si photodetector are 8.2 × 10^6^ and 1.35 × 10^13^ Jones, rendering it a desirable device for detecting weak signals with high sensitivity. The formulas for calculating the performance parameters and the detailed values under different conditions are summarized in the Supplementary Information.

Furthermore, the practical suitability of the Gr/GdIG/Si Schottky junction for high-performance weak-light photodetection was studied. Figure 4c provides large-scale power-dependent measurements with six orders of magnitude at a wavelength of 633 nm. The currents exhibit a monotonic increase when enlarging the illumination power densities. Typically, we can see the rise of photocurrent as the intensity increases slightly from dark to 5.2 nW/cm^2^, indicating the good weak-light detection competence of the proposed photodetector. It is observed that the photoresponse displays a near-linear variation concerning the increasing magnitudes at high intensities. Thus, we further verified this linearity with small steps of power variation between 10-30 mW/cm^2^, as shown in Fig. 4d. This demonstrates a stable operation capability in a wide range of light intensities. In addition, given that the detectable wavelength range is also important to a photodetector, we evaluated the broadband sensing competence between 230-950 nm using slightly changed microwatt-scale power densities. As plotted in Fig. 4e, the intensification of the current at each wavelength reveals a multiband operation covering the UV to NIR range, and the calculated D* and NEP values at a −2 V bias are presented in Fig. 4f, which shows a peak sensitivity at approximately 890 nm. Considering that the photoexcitation of this device mainly resides in silicon^8^, the reason for the peak sensitivity might be the typical absorption spectrum of silicon. This behavior demonstrates a broadband photoresponse of weak signals.

**Fig. 5:**
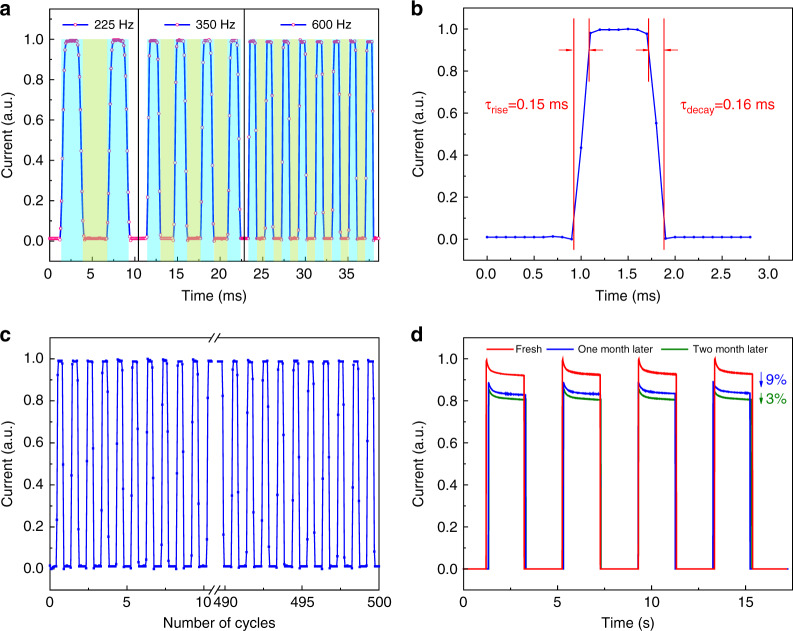
Response speed and stability characteristics of the Gr/GdIG/Si photodetector. **a** Photocurrent under varying light frequencies. **b** Transient response and recovery time. **c** Stability and repeatability test during 500 continuous ON/OFF cycles with a period of 10 seconds. **d** Stability measurements when exposed to air after one month and two months

Afterward, we characterized the temporal response properties of the Gr/GdIG/Si photodetector under pulsed illumination. Figure 5a presents the normalized currents generated by the transient 633 nm laser with frequencies of 225 Hz, 350 Hz, and 600 Hz. The device shows good resolution for on-off light switching, and the periodic rising and falling edges indicate that the currents originate from photoexcitation. The response time, defined as an increase in the photocurrent from 10% to 90%, is observed to be 0.15 ms, while the recovery time, defined analogously, is 0.16 ms (Fig. 5b). In addition, the working performance over multiple cycles was also identified. As plotted in Fig. 5c, the photocurrent and dark current are almost constant during 500 continuous operation cycles with a period of 10 seconds. Considering that the long-term stability of a Gr/Si photodetector has always been a challenge even in a dry box owing to the continuous growth of natural oxide on the surface of silicon^11^, the performance after long-term storage in air was tested. As illustrated in Fig. 5d, the device maintains proper detection ability after one month with a relatively small photocurrent decay of approximately 9%, followed by another 3% after the second month, demonstrating better environmental and time stability than our previous solution-based interface^12^. This may benefit from the good chemical stability and controlled thickness of the GdIG interlayer that prevents the growth of native oxide, but the still existing degradation can be attributed to the absorption of the water molecules and the atmospheric molecules in air. These impurities not only have a doping effect on graphene but are also harmful to the interface states of the junction^10,19^.

**Fig. 6:**
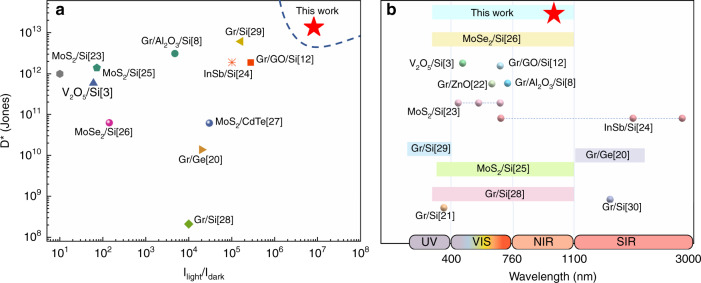
Performance comparison between the proposed Gr/GdIG/Si photodetector with similar structures. **a** specific detectivity and I_light_/I_dark_ ratio. **b** sensitive spectrum

In summary, the Schottky photodetector with a GdIG interlayer shows superior photoresponse behaviors, such as high specific detectivity (>10^13^), a large light/dark current ratio (>10^6^), broadband weak-light absorption, and good stability. A comparison is made between previously reported Schottky junction photodetectors with different interlayers, substrates, 2D materials, or basic structures^3,8,12,20–30^. This novel photodetector exhibits the advantages of high specific detectivity and a large light/dark current ratio at 633 nm under zero bias (Fig. 6a), and the sensitive spectrum remains at the upper-middle level (Fig. 6b). This is particularly encouraging, as only a very thin GdIG film is employed. These fascinating characteristics verify that the Gr/GdIG/Si Schottky junction can work as a high-quality photodetector and may hold dominance in next-generation light-sensing applications.

## Conclusion

This study demonstrates that a thin-layer GdIG film can serve as an interlayer of a graphene/silicon Schottky junction photodetector with remarkably improved performance owing to the effective increase in the Schottky barrier height and passivation of the contact surface. The photoresponse measurements at a −2 V bias show that the dark current of the photodetector decreases by 54 times, while the photocurrent increases slightly. More importantly, this novel structure exhibits high performance in a self-powered mode in terms of an I_light_/I_dark_ ratio up to 8.2 × 10^6^ and a specific detectivity of 1.35 × 10^13^ Jones, demonstrating remarkable advantages in weak-light detection. Further practicability characterizations reveal a broadband absorption spectrum covering UV to NIR, a near-linear response with a wide range of light intensities, and a speed of 0.15 ms. The device also exhibits a stable response for 500 continuous ON/OFF cycles and long-term environmental stability after several months. The intrinsic mechanism after inserting the interfacial layer has been explored through thermionic emission theory and the energy band structure. The decreased reverse saturation current and the increased Schottky barrier height play a dominant role in this development, which suppresses the thermally generated chargers and facilitates the transport of photogenerated carriers. These findings reveal the good prospects of GdIG film as an interface engineering material and provide a new approach for high-performance Gr/Si Schottky photodetectors.

## Supplementary information

Supplementary Information
