# Supplementary material for: High-performance photodetector based on an interface engineering-assisted graphene/silicon Schottky junction
Source: Microsyst Nanoeng. 2022 Jan 7;8:9. doi: 10.1038/s41378-021-00332-4 (PMC8741776; doi:10.1038/s41378-021-00332-4)
Supplement: Supplementary file 1 — Supplementary Information [file 41378_2021_332_MOESM1_ESM.docx]

**Supplementary Information**

High-Performance Photodetector based on Interface Engineering-Assisted Graphene/Silicon Schottky Junction

Peirui Ji^1^, Shuming Yang^1*^, Yu Wang^2^, Kaili Li^2^, Yiming Wang^1^, Hao Suo^1^, Yonas Tesfaye Woldu^1^, Xiaomin Wang^1^, Fei Wang^1^, Liangliang Zhang^1^, Zhuangde Jiang^1^

^1^State Key Laboratory for Manufacturing Systems Engineering, International Joint Laboratory for Micro/Nano Manufacturing and Measurement Technologies, Xi’an Jiaotong University, Xi’an 710049, China.

^2^MOE Key Laboratory for Nonequilibrium Synthesis and Modulation of Condensed Matter, Xi’an Jiaotong University, Xi’an, 710049, China.

*Corresponding Author

Email: shuming.yang@mail.xjtu.edu.cn

**1.** **Growth of GdIG thin film**

Gadolinium Iron Garnet (Gd_3_Fe_5_O_12_, GdIG) target was prepared firstly by the two-step solid-phase sintering method. After weighing according to the stoichiometry, gadolinium oxide (Aladdin, 99.99%) and iron oxide (Aladdin, 99.99%) powder were ball-milled, dried, and pre-sintered (1150 ℃ for 5 h). Afterward, the mixture was ground to a fine powder, ball-milled a second time, and pressed into a round shape with a diameter of 50.1 mm, a thickness of 2 mm, and then sintered again under 1350 °C, holding 10 h. With this target, the insulator GdIG garnet film was grown via RF magnetron sputtering technology on the exposed silicon window with precisely controlled thickness. The sputtering power was set to 60 W, and the pressure was fixed on 1 Pa. During the sputtering process, oxygen and argon were introduced at a ratio of 1:1. The thickness of the film was calibrated by atomic force microscopy to adjust the sputtering rate.

**2.** **Synthesis of monolayer graphene**

The chemical vapor deposition (CVD) method was adopted to synthesis graphene membrane on copper foil followed by spin-coating of polymethyl methacrylate (PMMA) as a support layer, and the copper foil was then etched off using iron trichloride (FeCl_3_) solution to obtain the graphene/PMMA film for later use.

**3. Characterization Methods**

The morphology of the GdIG thin film was identified via atomic force microscopy (Bruker, Innova). X-ray photoelectron spectrometer (Thermo Fisher, ESCALAB Xi+) was utilized to characterize the chemical composition of the as-prepared GdIG interlayer. Raman scattering measurement (HORBIA Jobin Yvon LabRAM, HR800) was carried out at a wavelength of 532 nm to study the quality of monolayer graphene. The surface topography of the Gr/GdIG/Si Schottky junction was observed using an optical microscope (Keyence, VHX-600). Electrical and photoresponse characteristics were measured via a semiconductor parameter analyzer (Keysight, B1500A). The broadband spectrum was produced by a xenon lamp (Spectral Products, ASB-XE-175-BFEX) combined with a monochromator (Spectral Products, CM110). The pulsed illumination was generated through a mechanical chopper placed in front of the light source.

**4. XPS spectra of Gd 4d and Fe 2p**

Fig. S1 plots the detailed XPS spectra of Gd 4d and Fe 2p of the as-grown GdIG film. The Gd 4d core levels are split into Gd 4d_5/2_ and Gd 4d_3/2_ owing to the spin-orbit coupling, while Fe 2p core levels are divided into Fe 2p_3/2_ and Fe 2p_1/2_ as well. The peaks of binding energies at 140.6 and 146.5 eV in Fig. S2a represent Gd 4d_5/2_ and Gd 4d_3/2_, while the peaks in Fig. S2b correspond to Fe 2p_3/2_ and Fe 2p_1/2_ respectively, which are consistent with the previously reported results^1^. Based on the peak area and corresponding sensitivity factors, the ratio of Gd to Fe is calculated to be approximately 3:5, and the O element is slightly higher than the chemical formula due to the adsorption of oxygen and water vapor from the air^2^.


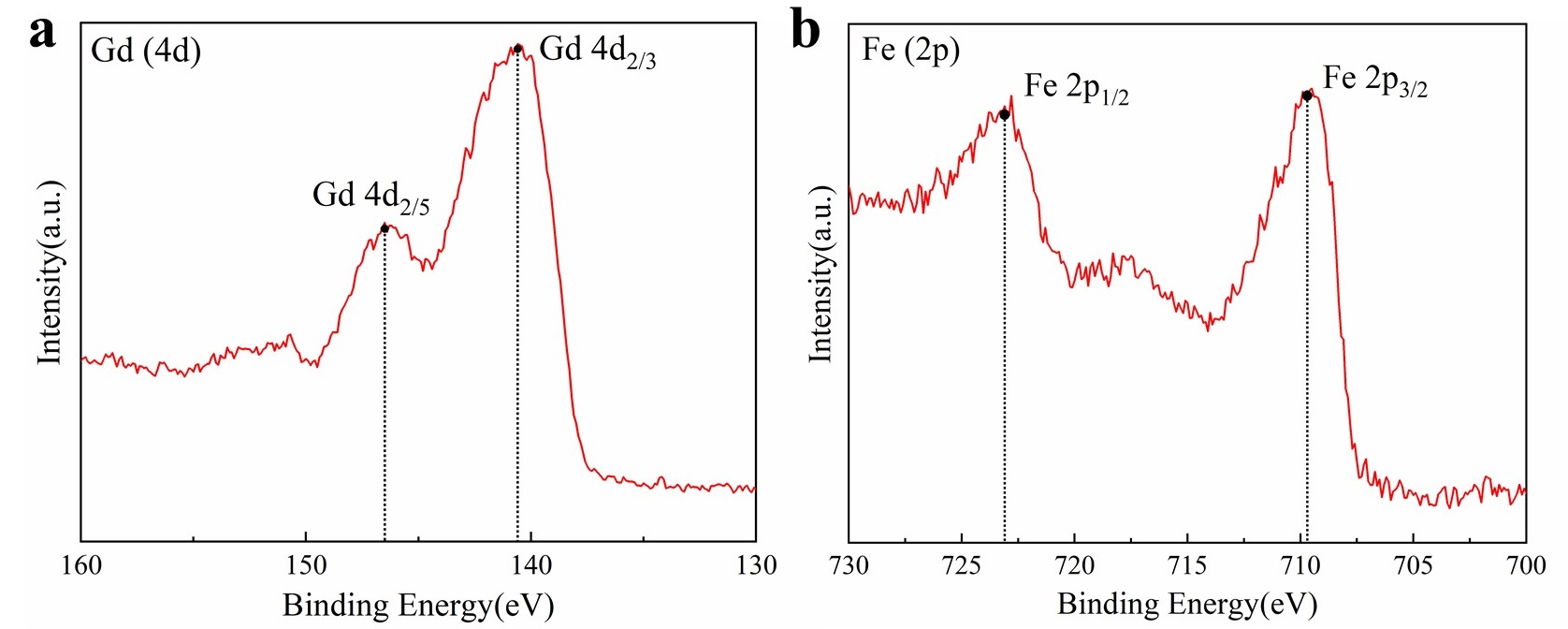


**Fig. S1.** XPS spectra of the as-grown GdIG film. (a) Gd 4d, (b) Fe 2p.

**5. Sample statistics**

In this study, two groups of ten samples were fabricated using the same process, of which five contained interlayers. The results of each group were approximately the same. The dark current values at -2V bias are summarized in the following tables. As can be seen, devices with interlayers generally have lower dark current and each group exhibits similar order of magnitudes, thus we took the first couple for in-depth study.

**Table S1.** **The dark current** **statistics of Gr/Si photodetectors**

| Device | Gr/Si-1 | Gr/Si-2 | Gr/Si-3 | Gr/Si-4 | Gr/Si-5 |
| --- | --- | --- | --- | --- | --- |
| I_dark_(nA) | 127 | 113 | 98.2 | 113 | 120 |

**Table S2.** **The dark current statistics of Gr/GdIG/Si photodetectors**

| Device | Gr/GdIG/Si-1 | Gr/GdIG/Si-2 | Gr/GdIGSi-3 | Gr/GdIGSi-4 | Gr/GdIG/Si-5 |
| --- | --- | --- | --- | --- | --- |
| I_dark_(nA) | 2.35 | 1.95 | 1.53 | 4.56 | 2.87 |

**6. Calculation of Schottky junction parameter**

According to the most commonly used thermionic emission theory^3^, the electrical transport properties of a Schottky diode under dark conditions can be described by,

| $I=I_{0}(e^{\frac{qV}{\eta kT}}\text{-1})$ | (1) |
| --- | --- |

where *q*, *η*, *k*, and *T* are the electronic charge, ideality factor, Boltzmann constant, and the temperature, respectively. *I_0_* is the reverse saturation current,

| $I_{0}=AA^{*}T^{2}e^{(-\frac{\Phi_{B}}{kT})}$ | (2) |
| --- | --- |

where *A*=0.0256 cm^2^ is the photosensitive area, *A** =112 A cm^–2^K^–2^ is the Richardson coefficient of n-Si.

When the forward bias voltage satisfies V>3kT/q, Eq. (1) can be approximated as,

| $I=I_{0}(e^{\frac{qV}{\eta kT}})$ | (3) |
| --- | --- |

Through a simple linear fitting of Eq. (3) to ln(I)-V^4^, the ideality factor and reverse saturation current can be extracted by the slope and intercept of the fitting curve. Based on these, the Schottky barrier height can be calculated by Eq. (2). The parameter values of both Gr/Si and Gr/GdIG/Si junctions are summarized in the following table.

**Table S3.** **Schottky junction** **parameter summary of the photodetectors**

| Parameter  Device | Ideality factor | Reverse saturation current  (nA) | Schottky barrier height  (eV) |
| --- | --- | --- | --- |
| Gr/Si | 3.1 | 6.5 | 0.81 |
| Gr/GdIG/Si | 2.0 | 0.72 | 0.87 |

**7. Calculation of photoresponse parameter**

The ON/OFF ratio of a photodetector is defined as the photo/dark current ratio. Responsivity (*R*) is calculated by the ratio of the photocurrent to the light intensity^5^,

| $R=\frac{I_{ph}}{P}=\frac{I_{ph}}{P_{opt}S}$ | (4) |
| --- | --- |

where *I_ph_* is the photocurrent, defined as the difference between the total current under illumination and the dark current. *P* is the illumination power, defined as the product of power density (*P_opt_*) and irradiation area (*S*).

Specific detectivity (*D**) and noise equivalent power (*NEP*) reflect the limit of weak light detection, which are defined as^6,7^,

|  | (5) |
| --- | --- |
| $NEP=\frac{\sqrt{A}}{D^{*}}$ | (6) |

where *A* is the device photosensitive area, *q* is the electronic charge, and *I_dark_* is the dark current. In this work, the values of *S*=0.0192 cm^2^ and *A*=0.0256 cm^2^.

Based on these formulas, the performance parameters at 633 nm illumination with a power density of 60 mW/cm^2^ are calculated and summarized as follows.

**Table S4.** **Photoresponse parameter summary of the photodetectors**

| Parameter  Device | I_dark_  (nA) | I_ph_  (mA) | R  (A/W) | ON/OFF ratio | D*  (Jones) | NEP  (pW/Hz^1/2^) |
| --- | --- | --- | --- | --- | --- | --- |
| Gr/GdIG/Si,  0 V bias | 0.045 | 0.37 | 0.32 | 8.2×10^6^ | 1.35×10^13^ | 0.01 |
| Gr/GdIG/Si,  -2 V bias | 2.35 | 1.04 | 0.90 | 4.4×10^5^ | 5.25×10^12^ | 0.03 |

**References**

^1^ Mathur, S. *et al.*, Nanocrystalline orthoferrite GdFeO3 from a novel heterobimetallic precursor. *ADV MATER* **14** 1405 (2002).

^2^ Soderlind, F., Selegard, L., Nordblad, P., Uvdal, K. & Kall, P., Sol-gel synthesis and characterization of polycrystalline GdFeO3 and Gd3Fe5O12 thin films. *J SOL-GEL SCI TECHN* **49** 253 (2009).

^3^ CHEUNG, S. & CHEUNG, N., Extraction of Schottky diode parameters from forward current‐voltage characteristics. *APPL PHYS LETT* **49** 85 (1986).

^4^ Guo, H. *et al.*, Silicon- and oxygen-codoped graphene from polycarbosilane and its application in graphene/n-type silicon photodetectors. *APPL SURF SCI* **464** 125 (2019).

^5^ Cheng, Y. *et al.*, Photovoltaic broadband photodetectors based on CH3NH3PbI3 thin films grown on silicon nanoporous pillar array. *SOL ENERG MAT SOL C* **204** 330 (2020).

^6^ Wang, Y., Yang, S., Lambada, D. R. & Shafique, S., A graphene-silicon Schottky photodetector with graphene oxide interlayer. *SENSOR ACTUAT A-PHYS* **112232** (2020).

^7^ Li, X. *et al.*, High Detectivity Graphene-Silicon Heterojunction Photodetector. *SMALL* **12** 595 (2016).
